# Cardiac arrhythmia and epilepsy genetic variants in sudden unexpected death in epilepsy

**DOI:** 10.3389/fneur.2024.1386730

**Published:** 2024-05-02

**Authors:** Amir Aschner, Anne Keller, Andrew Williams, Robyn Whitney, Kris Cunningham, Robert M. Hamilton, Michael Pollanen, Elizabeth Donner

**Affiliations:** ^1^Division of Neurology, Hospital for Sick Children, University of Toronto, Toronto, ON, Canada; ^2^Department of Laboratory Medicine and Pathobiology, Temerty Faculty of Medicine, University of Toronto, Toronto, ON, Canada; ^3^McMaster Children’s Hospital, McMaster University, Hamilton, ON, Canada; ^4^Department of Pathology and Molecular Medicine, School of Medicine, Faculty of Health Sciences, Queen’s University, Kingston, ON, Canada; ^5^Division of Cardiology, Hospital for Sick Children, University of Toronto, Toronto, ON, Canada; ^6^Department of Pediatrics, Faculty of Medicine, University of Toronto, Toronto, ON, Canada

**Keywords:** SUDEP (sudden unexpected death in epilepsy), cardiac arrhythmias, biomarker, population allele frequencies, gene panel

## Abstract

**Introduction:**

Sudden Unexpected Death in Epilepsy (SUDEP) is the leading epilepsy-related cause of death, affecting approximately 1 per 1,000 individuals with epilepsy per year. Genetic variants that affect autonomic function, such as genes associated with cardiac arrhythmias, may predispose people with epilepsy to greater risk of both sudden cardiac death and SUDEP. Advances in next generation sequencing allow for the exploration of gene variants as potential biomarkers.

**Methods:**

Genetic testing for the presence of cardiac arrhythmia and epilepsy gene variants was performed via genetic panels in 39 cases of SUDEP identified via autopsy by the Ontario Forensic Pathology Service. Variants were summarized by in-silico evidence for pathogenicity from 4 algorithms (SIFT, PolyPhen-2, PROVEAN, Mutation Taster) and allele frequencies in the general population (GnomAD). A maximum credible population allele frequency of 0.00004 was calculated based on epilepsy prevalence and SUDEP incidence to assess whether a variant was compatible with a pathogenic interpretation.

**Results:**

Median age at the time of death was 33.3 years (range: 2, 60). Fifty-nine percent (*n*=23) were male. Gene panels detected 62 unique variants in 45 genes: 19 on the arrhythmia panel and 26 on the epilepsy panel. At least one variant was identified in 28 (72%) of decedents. Missense mutations comprised 57 (92%) of the observed variants. At least three *in silico* models predicted 12 (46%) cardiac arrhythmia panel missense variants and 20 (65%) epilepsy panel missense variants were pathogenic. Population allele frequencies were <0.00004 for 11 (42%) of the cardiac variants and 10 (32%) of the epilepsy variants. Together, these metrics identified 13 SUDEP variants of interest.

**Discussion:**

Nearly three-quarters of decedents in this SUDEP cohort carried variants in comprehensive epilepsy or cardiac arrhythmia gene panels, with more than a third having variants in both panels. The proportion of decedents with cardiac variants aligns with recent studies of the disproportionate cardiac burden the epilepsy community faces compared to the general population and suggests a possible cardiac contribution to epilepsy mortality. These results identified 13 priority targets for future functional studies of these genes potential role in sudden death and demonstrates the necessity for further exploration of potential genetic contributions to SUDEP.

## Introduction

Sudden death affects the epilepsy population at a rate 24 times the general population (1, 2), and sudden unexpected death in epilepsy (SUDEP) is the leading epilepsy-related cause of death, affecting approximately 1 per 1,000 individuals with epilepsy each year (3–5). SUDEP refers to deaths in people with epilepsy (PwE) without a known cause and is usually accompanied by an autopsy that identifies no anatomical or toxicological cause of death (6). SUDEP rates are higher in certain epilepsy groups, including those with poorly controlled seizures, epilepsy surgery candidates, and some genetic epilepsies, such as Dravet Syndrome (DS) (7, 8).

Understanding the pathophysiology of SUDEP is a top priority among people living with epilepsy, caregivers, and researchers. The United States National Institute of Neurological Disorders and Stroke (NINDS) released epilepsy research guidelines in 2007 which included the goals of identification of biomarkers for epileptogenesis for risk stratification, and identification of the mechanisms responsible for SUDEP (9). In 2022, the Ontario Brain Institute (OBI), in partnership with the Epilepsy Research Program (EpLink), published the top 10 unanswered questions in epilepsy according to patients, caregivers, and healthcare providers across Canada. The top priority related to the identification of genetic biomarkers to diagnose and treat epilepsy, and the fourth pertained to risk factors for SUDEP.^1^ The identification of biomarkers and risk factors for SUDEP is critical to providing appropriate care, as well as understanding how SUDEP risk can be mitigated.

Growing evidence implicates autonomic dysregulation (10, 11), especially cardiorespiratory dysfunction (12–14) in the mechanism of SUDEP, however, risk factors usually associated with these events have not been definitively linked to SUDEP. Despite this, several clinical risk factors for SUDEP have been identified, with the presence and frequency of bilateral tonic–clonic seizures (BTC) being the most robust (15–18). Other risk factors include nocturnal seizures, living alone or not sharing a bedroom, and failure to optimize treatment with anti-seizure medications (18–20). Despite best efforts to identify those at high risk of SUDEP, it is still not understood why many people at high risk will live for years with frequent BTC while others may die after just one or two seizures. The insufficiency of clinical risk factors emphasizes the need for the identification of different biomarkers to identify PwE at the greatest risk for SUDEP. Reliable biomarkers may inform future studies and effective SUDEP prevention strategies.

Cardiac arrhythmia genes are of particular interest as potential biomarkers given growing evidence for heart-brain interactions (21, 22) and similarities in the characteristics of SUDEP and sudden cardiac death (23, 24). Multiple genes have been identified that are associated with arrhythmogenic epilepsy; single gene variants that produce both epilepsy and cardiac arrhythmias (21, 25, 26). Many genes that produce epilepsy and cardiac phenotypes encode ion channels which are often expressed in both heart and brain tissue. Genetic epilepsies associated with channelopathies often have higher SUDEP rates (27), which suggests factors specific to channel disorders may have a role in SUDEP pathogenesis (28). Even when variants in cardiac genes are not associated with epilepsy or produce a cardiac phenotype in isolation, there is the possibility of polygenic risk whereby the presence of multiple gene variants in an individual confers an additive or multiplicative effect on SUDEP risk compared to one gene alone.

In humans, sudden cardiac death occurs 2.8 times more frequently in PwE compared to the general population (29), and a disproportionate number of PwE and individuals from SUDEP cohorts were found to have pathogenic variants in cardiac genes (30–34). Altered cardiac function has been observed in animal models of DS (35, 36) and *SCN8A*-related developmental and epileptic encephalography (DEE) (37) with cardiac arrhythmias often preceding SUDEP in these models. Likewise, seizures and epilepsy phenotypes have been observed in conjunction with sudden cardiac death in mouse models of Long QT Syndrome (38) and Catecholaminergic Polyventricular Tachycardia (39, 40).

Given the evidence of cardiac comorbidities among PwE and previous SUDEP cohorts, damaging cardiac gene variants are promising potential SUDEP biomarkers. We investigated the yield of cardiac- and epilepsy-related gene panels in a series of 39 SUDEP cases to explore whether those who die of SUDEP may be predisposed to sudden death by underlying genetic variants.

## Methods

This study was approved by the Hospital for Sick Children Research Ethics Board.

### Study cohort

The Ontario Forensic Pathology Service (OFPS) is tasked with investigation of all sudden or unexpected deaths in Ontario, Canada, which typically includes autopsy, toxicology investigation, and when appropriate genetic testing. This study retrospectively reviewed reports of SUDEP identified by the OFPS between January 2014 – June 2016. The OFPS shared the case descriptions of 83 decedents. Two neurologists with epilepsy subspecialty training and SUDEP research expertise reviewed the cases for SUDEP criteria and identified 39 cases with Definite or Definite Plus SUDEP classifications (6).

Data collected included case details, toxicology report, and genetic testing findings. Where available from the coroner’s warrant, a summary of the decedent’s medical history, medications, and circumstances surrounding the death were extracted from the autopsy reports.

### Genetic analysis

DNA samples were extracted post-mortem from blood or frozen tissue. No samples from family members were provided. Next generation sequencing was performed by GeneDx and included the Comprehensive Arrhythmia Panel and the Comprehensive Epilepsy Panel including deletion/duplication analysis. At time of sequencing, the epilepsy and arrhythmia panels included 87 and 46 genes, respectively.

Complete coding regions and splice site junctions of the genes tested were enriched using a proprietary targeted capture system developed by GeneDx. Enriched targets were sequenced with paired-end reads on an Illumina platform. Bi-directional sequence reads were assembled and aligned to reference sequences based on NCBI RefSeq transcripts and human genome build GRCh37/UCSC hg19. After gene-specific filtering, data were analyzed to identify sequence variants and most deletions and duplications involving coding exons; however, technical limitations and inherent sequence properties effectively reduce this resolution for some genes. Alternative sequencing or copy number detection methods were used to analyze or confirm regions with inadequate sequence or copy number data by next generation sequencing.

Identified variants were classified according to American College of Medical Genetics and Genomics and the Association for Molecular Pathology (ACMG/AMP) guidelines (41), as well as by ClinVar^2^ interpretation, by pathogenicity predictions from four *in-silico* algorithms [SIFT^3^ (42), PROVEAN v1.1.3^4^ (43), PolyPhen-2^5^ (44), and MutationTaster^6^ (45)], and by population allele frequencies in the general population. Allele frequencies were calculated as the total group maximum filtering allele frequency in exomes and genomes reported by GnomAD v4.0.0.^7^ All data were retrieved January 10, 2024.

We used the frequency-based framework outlined by (46) to assess whether a variant was “too common” to be considered pathogenic; Assuming an epilepsy prevalence of 5/1000 (47) and a SUDEP incidence of 1/1000 per year (4, 5) we calculated a maximum credible population allele frequency of 0.00004. Variants that were predicted to be pathogenic by at least three *in-silico* algorithms and occurred below the population allele frequency threshold were classified as SUDEP variants of interest (VoI).

### Statistical analyses

Continuous variables were compared between groups using Wilcoxon ranked sum test with continuity correction. Categorical variables were compared with Fisher’s exact test. All statistical analyses were performed using R (version 3.2.1.), and a *p* < 0.05 was used to indicate statistical significance. Percentages reported for clinical parameters are based on non-missing data.

## Results

### Cohort characteristics

The SUDEP cohort consisted of 39 decedents. These SUDEP cases were included in a previous report on cause of death in PwE (48). All cases were classified as Definite or Definite Plus SUDEP. Median age at time of death was 33 years (IQR:18–49) and ranged from 2 to 60 years. Males accounted for 59% (*n* = 23). A qualitative summary of the cohort is presented in Table 1.

**Table 1:** Demographic and clinical characteristics of the DS cohort.

Variable	All cases (N=39)	With Variants of Interest (n=11)	Without Variants of Interest (n=28)	Comparison
*Age at death* Median (IQR); Range	37 (18-49); 2-60	38 (18-49); 4-60	35.5 (18.5-48.25); 2-60	p=0.89
*Male sex*, no. (%)	23 (59%)	4 (36%)	19 (68%)	p=0.14
*Seizure frequency in preceding month(s)* Well-controlled Poorly controlled Unknown	7 (41%) 10 (59%) 22	1 (25%) 3 (75%) 7	6 (46%) 7 (54%) 15	p=0.60
*Anti-Seizure Medications* None Single Multiple Unknown	5 (16%) 13 (42%) 13 (42%) 8	1 (10%) 5 (50%) 4 (40%) 1	4 (19%) 8 (38%) 9 (43%) 7	p=0.88
*Recent change in epilepsy status* Yes No	5 (13%) 34 (87%)			
Comorbidities* Acute Cardiac Mental health Metabolic Neurologic Non-medical drug use Pulmonary Trauma	Recent illness (n=13) Atherosclerosis (n=3), High blood pressure/cholesterol, Hypertension Depression (n=4), Schizoaffective disorder, Schizophrenia (n=2) Diabetes (n=2), Hypothyroidism, Obesity (n=4) Angelman Syndrome, Autism, Cerebral palsy (n=4), Cortical dysplasia, Dementia, Developmental delay (n=4), Fibromyalgia, Multiple sclerosis, Parkinson’s disease, Stroke Alcohol (n=3), Cannabis (n=3), Cigarettes (n=3), Other (n=2) Asthma (n=2), Chronic obstructive pulmonary disease, Recurrent aspiration pneumonia, Chronic bronchitis History of head injury (n=3)			

*n=1 unless otherwise stated; Counts within categories are not necessarily mutually exclusive.

Seizure frequency data were available for 17 cases and reported as well-controlled (i.e., seizure free or infrequent) in 7 cases (41%) and poorly controlled (i.e., multiple per month) in 10 cases (59%). Data for anti-seizure medications (ASM) were available in 31 cases, with zero ASM reported actively taken in five cases (16%), a single ASM in 13 cases (42%), and two or more ASM in 13 cases (42%). The most frequently reported ASM were phenytoin (*n* = 8), valproate (*n* = 8), and carbamazepine (*n* = 5). Seizure status had recently changed for five (13%) cases, including one increase in seizure frequency and four who had weaned or changed their ASM. Toxicology and post-mortem reports indicated that three decedents were potentially non-adherent with ASM treatment.

Comorbidities were present in at least 29 cases (74%). The most common categories of comorbidities reported were neurologic (e.g., cerebral palsy), substance misuse, and acute (e.g., recent illness). Other comorbidities fell under cardiac, mental health, metabolic, pulmonary, and trauma categories (Table 1). Additional relevant medical history was limited; however, autopsy reports noted the following pertinent to cardiac history: one decedent experienced cardiac arrest after a seizure earlier in the year, and one decedent had a father who died of myocardial infarct. Lastly, one case experienced respiratory arrest after a seizure earlier in the year. All autopsy reports detailed unremarkable cardiac findings.

### Sudden unexpected death in epilepsy characteristics

Circumstances of death are presented in Table 2. The location where the deceased was found was reported for 38 cases (97%). Most individuals (*n* = 27, 71%) were found in a bed – their own, a caregiver’s, or otherwise. Others were found in a bathroom (*n* = 3, 8%), elsewhere in their home (*n* = 7, 18%), or outside (*n* = 1, 3%). Body position was reported for 21 cases: 12 prone (57%), seven supine (33%), and two side-lying (10%). Evidence of a seizure immediately preceding SUDEP was reported for 25 cases. Examples of accepted evidence included a witnessed seizure, tongue bites, vomit, frothy fluid at the mouth, and found in an awkward position suggestive of recent convulsion. Only one case explicitly reported there was no evidence of a seizure; the remainder were unknown (*n* = 13). Only two deaths were witnessed, while 37 (95%) were unwitnessed.

**Table 2:** SUDEP characteristics of the DS cohort.

Variable	n (%)
Location Bed/bedroom Bathroom/sauna Home, other Outside Unknown	27 (71%) 3 (8%) 7 (18%) 1 (3%) 1
Position Prone Supine Side lying Unknown	12 (57%) 7 (33%) 2 (10%) 18
Evidence of a seizure Yes No Unknown	25 (96%) 1 (4%) 13
Witnessed Yes No	2 (5%) 37 (95%)

Data on epilepsy diagnosis, etiology, and years lived with epilepsy were not available from autopsy reports. Limited data was available regarding seizure semiology and prescription drug use.

### Gene panel findings

Genetic testing revealed 62 unique variants detected in 45 genes: 19 genes from the arrhythmia panel and 26 from the epilepsy panel. A full list of both panel’s genes tested is available in Supplemental Table S1. At least one variant was identified on either the epilepsy or cardiac panel in 28 (72%) decedents. Among those with at least one variant, 21 (54%) had variants in genes listed on the epilepsy panel and 21 had genes listed on the cardiac panel. A single variant was identified in 10 (36%) cases, while two or more variants were found in 18 (64%) cases. Among those with multiple variants, eight (21%) had multiple cardiac variants, six (15%) had multiple epilepsy variants, and 14 (36%) had at least one variant in both panels (Table 3). Missense variants comprised 57 (92%) of the observed variants. Other variant types included duplication of an exon (*n* = 1), deletion of an exon (*n* = 1), intronic variants (*n* = 2), and variants in untranslated regions (*n* = 1).

**Table 3:** Overview of genetic findings by decedent.

Subject ID	Age	Arrhythmia Panel			Epilepsy Panel		
		Gene	Nucleotide Change	Amino Acid Change	Gene	Nucleotide Change	Amino Acid Change
01	40	*SNTA1*	c.1517_*2dupAGAA	N/A	*PNKP*	c.650C>G	(p.Thr217Ser)
03	38	*SCN5A*	c.5286C>G	(p.Ile1762Met)	*CACNA1A* *CHRNA4* *CHRNA4* *CHRNA4* *CHRNA4*	c.2407C>A c.1531G>T c.1421C>G c.1012C>G c.560C>G	(p.Arg803Ser) (p.Ala511Ser) (p.Pro474Arg) (p.His338Asp) (p.Ala187Gly)
04	49	*SCN10A* *KCNE1* *ANK2*	c.1489C>T c.193T>C c.11716C>T	(p.Arg497Cys) (p.Tyr65His) (p.Arg3906Trp)	Negative		
07	34	Negative			*PIGO*	c.253C>A	(p.Pro85Thr)
08	54	*SNTA1*	c.1015C>T	(p.Arg339Cys)	Negative		
09	57	*RYR2* *TRPM4*	c.2402G>T Deletion of exon 20	(p.Arg801Leu)	*WWOX*	c.1044C>A	(p.Phe348.Leu)
	41	*TRDN* *KCNJ8*	c.1367A>G c.1055G>A	(p.Gln456Arg) (p.Arg352Gln)	*GOSR2* *PIGV*	c.509A>G c.348_349delGAinsAG	(p.Asn170Ser) (p.Ile117Val)
11	26	Negative			*SLC13A5*	c.862G>A	(p.Gly288Arg)
14	5	*LMNA*	c.998 C>G	(p.Thr333Ser)	*MFSD8*	c.818A>G	(p.Asn273Ser)
15	5	Negative			*PIGA*	c.613G>A	(p.Val205Ile)
17	49	*RANGRF*	c.52C>T	(p.Leu18Phe)	*PIGO*	c.3163T>C	(p.Phe1055Leu)
18	4	*SCN10A*	c.3803G>A	(p.Arg1268Gln)	*EPM2A*	c.487A>G	(p.Asn163Asp)
19	28	*LMNA*	c.1930C>T	(p.Arg644Cys)	*SCN1A*	c.5422T>C	(p.Phe1808Leu)
20	5	*TGFB3*	c.412T>G	(p.Ser138Ala)	*PNKP*	c.1397T>C	(p.Met466Thr)
21	37	*RYR2*	c.8162T>C	(p.Ile2721Thr)	Negative		
22	26	Negative			*SCARB2*	c.194A>G	(p.Tyr65Cys)
23	8	*DSG2* *DSC2* *TTN*	c.2434G>T c.1787C>T c.34858G>T	(p.Gly812Cys) (p.Ala596Val) (p.Val11620Leu)	*CTSD*	c.8C>T	(p.Pro3Leu)
24	52	Negative			*CHRNA2*	c.140C>T	(p.Thr47Met)
27	37	Negative			*PIGA*	c.517G>A	(p.Val173Met)
30	28	*JUP* *SCN10A*	c.1696G>A c.3803G>A	(p.Ala566Thr) (p.Arg1268Gln)	Negative		
32	60	*TTN* *SCN10A*	c.7057+2dupT c.3803G>A	IVS30+2dupT) (p.Arg1268Gln)	*GRIN2B* *SLC25A22*	c.3994G>T c.500G>A	(p.Asp132Tyr) (p.Arg167Gln)
35	44	Negative			*CDKL5*	c.2572C>T	(p.Arg858Cys)
39	54	*SCN10A*	c.5339C>T	(p.Pro1780Leu)	*ARX* *CHD2* *TBC1D24*	c.625G>C c.2505+4A>G c.169C>T	(p.Gly209Arg) IVS19+4A>G (p.Arg57Cys)
42	11	*DSG2*	c.46A>G	(p.Ile16Val)	Negative		
43	48	*SCN5A* *KCNQ1* *KCNQ1*	c.5803G>A c.328G>A c.217C>A	(p.Gly1935Ser) (p.Val110Ile) (p.Pro73Thr)	Negative		
46	49	*RYR2*	c.7495G>A	(p.Ala2499Thr)	*SCN2A* *GABRB2*	c.1858C>T c.9A>T	(p.Arg620Trp) (p.Arg3Ser)
47	15	*SCN2B* *CACNA1C*	c.625_626delAAINSCC Duplication of exons 1-11	(p.Asn209Pro) -	Negative		
48	31	*RYR2*	c.3257G>A	(p.Arg1086Gln)	*NRXN1* *POLG* *TPP1*	c.2653C>T c.803G>C c.1241A>T	(p.His885TYR) (p.Gly268Ala) (p.Asn404Ile)

According to the ACMG/AMP guidelines 1 (2%) variant was classified as pathogenic – *DSG2* c.2434G > T (p.Gly812Cys) – 4 (7%) as likely pathogenic, 5 (9%) as likely benign, and 47 (82%) as uncertain significance. Interpretations of the pathogenicity of the 57 missense variants by ClinVar were Benign or Likely Benign for five (9%), Variant of Uncertain Significance (VUS) for 27 (47%) and Conflicting Evidence for 25 (44%). Among the variants with Conflicting Evidence, 14 (56%) were predominantly Benign or Likely Benign. One likely pathogenic *EPM2A* variant associated with Lafora disease, and one likely pathogenic *SCN1A* variant associated with DS were identified on the epilepsy panel (Table 4).

**Table 4:** Summary of genetic variants identified in the SUDEP cohort. Four *in silico* algorithms were applied, PolyPhen2, PROVEAN, SIFT, and MutationTaster. Genes are organized alphabetically by panel. Population allele frequency threshold was predetermined to be 0.00004. Variants of Interest (VoI) are bolded.

Gene	dbSNP	ACMG/AMP classification	ClinVar classification	*in silico* algorithm predictions				No. of models predicting pathogenic	Below allele frequency threshold
				PolyPhen2	PROVEAN	SIFT	MutationTaster		
Cardiac genes									
*ANK2*	rs121912706	VUS	Conflicting	Probably damaging	Deleterious	Damaging	Disease causing	4	No
*CACNA1C*	-	VUS	VUS	-	-	-	-	-	
*DSC2*	rs148185335	VUS	Conflicting	Benign	Deleterious	Damaging	Polymorphism	2	No
*DSG2*	**rs121913010**	**Pathogenic**	**Conflicting**	**Probably damaging**	**Deleterious**	**Damaging**	**Disease causing**	**4**	**Yes**
*DSG2*	rs376660601	LB	VUS	Benign	Neutral	Tolerated	Polymorphism	0	Yes
*JUP*	-	VUS	VUS	Possibly damaging	Neutral	Tolerated	Disease causing	2	Yes
*KCNE1*	**-**	**VUS**	**VUS**	**Probably damaging**	**Deleterious**	**Damaging**	**Disease causing**	**4**	**Yes**
*KCNJ8*	rs747622709	VUS	VUS	Benign	Neutral	Tolerated	Disease causing	1	Yes
*KCNQ1*	rs199472676	LP	VUS	Benign	Neutral	Damaging	Polymorphism	1	No
*KCNQ1*	rs199472677	VUS	VUS	Benign	Neutral	Tolerated	Disease causing	1	No
*LMNA*	rs142000963	VUS	Conflicting	Possibly damaging	Neutral	Damaging	Disease causing	3	No
*LMNA*	-	VUS	VUS	Benign	Neutral	Tolerated	Disease causing	1	Yes
*RANGRF*	rs150856064	VUS	VUS	Benign	Deleterious	Tolerated	Disease causing	2	No
*RYR2*	**-**	**VUS**	**VUS**	**Probably damaging**	**Deleterious**	**Damaging**	**Disease causing**	**4**	**Yes**
*RYR2*	rs371157868	VUS	VUS	Probably damaging	Deleterious	Damaging	Disease causing	4	No
*RYR2*	**rs191850147**	**VUS**	**Conflicting**	**Probably damaging**	**Deleterious**	**Damaging**	**Disease causing**	**4**	**Yes**
*RYR2*	rs201500134	VUS	Conflicting	Probably damaging	Deleterious	Tolerated	Disease causing	3	No
*SCN10A*	rs370009920	VUS	Conflicting	Probably damaging	Deleterious	Damaging	Disease causing	4	No
*SCN10A*	**rs770288343**	**VUS**	**VUS**	**Probably damaging**	**Deleterious**	**Damaging**	**Disease causing**	**4**	**Yes**
*SCN10A*	rs138832868	VUS	Conflicting	Possibly damaging	Deleterious	Damaging	Disease causing	4	No
*SCN2B*	-	VUS	VUS	Unavailable	Neutral	Damaging	Disease causing	2	Yes
*SCN5A*	**-**	**VUS**	**VUS**	**Probably damaging**	**Neutral**	**Damaging**	**Disease causing**	**3**	**Yes**
*SCN5A*	rs199473637	LB	Conflicting	Benign	Neutral	Tolerated	Polymorphism	0	No
*SNTA1*	rs138164106	VUS	VUS	Probably damaging	Deleterious	Tolerated	Polymorphism	2	No
*SNTA1*	-	VUS	VUS	-	-	-	-	-	Not found
*TGFB3*	rs201453600	VUS	Conflicting	Benign	Neutral	Tolerated	Disease causing	1	No
*TRDN*	rs200243235	LB	Conflicting	Probably damaging	Neutral	Deleterious	Polymorphism	2	No
*TRPM4*	-	VUS	VUS	-	-	-	-	-	Not found
*TTN*	-	VUS	VUS	Probably damaging	Neutral	Tolerated	Polymorphism	1	Yes
*TTN*	rs765019023	VUS	VUS	-	-	-	-	-	Not found
Epilepsy genes									
*ARX*	rs587783203	VUS	VUS	Benign	Neutral	Tolerated	Polymorphism	0	No
*CACNA1A*	**rs760816963**	**VUS**	**Conflicting**	**Possibly damaging**	**Deleterious**	**Damaging**	**Disease causing**	**4**	**Yes**
*CDKL5*	**rs773760466**	**VUS**	**Conflicting**	**Unavailable**	**Deleterious**	**Damaging**	**Disease causing**	**3**	**Yes**
*CHD2*	rs767309501	VUS	Conflicting	-	-	-	-	-	Yes
*CHRNA2*	rs74772771	VUS	Conflicting	Benign	Neutral	Damaging	Polymorphism	1	No
*CHRNA4*	rs200069626	VUS	LB	Probably damaging	Deleterious	Damaging	Disease causing	4	No
*CHRNA4*	rs200197645	VUS	LB	Probably damaging	Deleterious	Tolerated	Disease causing	3	No
*CHRNA4*	rs199778549	LB	LB	Possibly damaging	Neutral	Damaging	Polymorphism	2	No
*CHRNA4*	rs200795334	LB	LB	Benign	Neutral	Tolerated	Polymorphism	0	No
*CTSD*	rs757712173	VUS	VUS	Unavailable	Neutral	Tolerated	Polymorphism	0	No
*EPM2A*	**rs777767978**	**LP**	**Conflicting**	**Benign**	**Deleterious**	**Damaging**	**Disease causing**	**3**	**Yes**
*GABRB2*	-	VUS	Benign/LB	Benign	Neutral	Tolerated	Disease causing	1	Yes
*GOSR2*	rs150907052	VUS	VUS	Possibly damaging	Deleterious	Tolerated	Disease causing	3	No
*GRIN2B*	**rs200421469**	**LP**	**VUS**	**Possibly damaging**	**Deleterious**	**Damaging**	**Not found**	**3**	**Yes**
*MFSD8*	**rs143288262**	**VUS**	**VUS**	**Probably damaging**	**Neutral**	**Damaging**	**Disease causing**	**3**	**Yes**
*NRXN1*	rs199784139	VUS	Conflicting	Probably damaging	Deleterious	Tolerated	Disease causing	3	No
*PIGA*	rs752395232	VUS	VUS	Unavailable	Neutral	Damaging	Disease causing	2	Yes
*PIGA*	-	VUS	VUS	Unavailable	Neutral	Damaging	Disease causing	2	No
*PIGO*	rs147316771	VUS	Conflicting	Probably damaging	Deleterious	Damaging	Disease causing	4	No
*PIGO*	rs745754359	VUS	VUS	Possibly damaging	Neutral	Tolerated	Disease causing	2	No
*PIGV*	-	VUS	Conflicting	Unavailable	Neutral	Tolerated	Disease causing	1	Not found
*PNKP*	rs145886749	VUS	VUS	Possibly damaging	Deleterious	Damaging	Disease causing	4	No
*PNKP*	rs115259839	VUS	Conflicting	Probably damaging	Deleterious	Tolerated	Disease causing	3	No
*POLG*	rs61752784	VUS	Conflicting	Probably damaging	Deleterious	Damaging	Disease causing	4	No
*SCARB2*	rs138955932	VUS	VUS	Probably damaging	Deleterious	Damaging	Disease causing	4	No
*SCN1A*	**-**	**LP**	**Pathogenic**	**Probably damaging**	**Deleterious**	**Damaging**	**Disease causing**	**4**	**Yes**
*SCN2A*	**rs762680220**	**VUS**	**VUS**	**Probably damaging**	**Deleterious**	**Damaging**	**Disease causing**	**4**	**Yes**
*SLC13A5*	rs761215437	VUS	VUS	Possibly damaging	Deleterious	Damaging	Polymorphism	3	No
*SLC25A22*	rs201089795	VUS	Conflicting	Benign	Neutral	Damaging	Disease causing	2	No
*TBC1D24*	rs202162520	VUS	Conflicting	Possibly damaging	Neutral	Damaging	Disease causing	3	No
*TPP1*	rs146798796	VUS	VUS	Probably damaging	Deleterious	Damaging	Disease causing	4	No
*WWOX*	rs1057524658	VUS	VUS	Probably damaging	Deleterious	Tolerated	Disease causing	3	No

*Annotations*: B, Benign; LB, Likely Benign; LP, Likely Pathogenic; VUS, Variant of Uncertain Significance.

The functional impact of each missense variant was predicted by four *in-silico* algorithms: PROVEAN, SIFT, PolyPhen-2, and MutationTaster (Table 4). These tools were selected as they are commonly used to interpret missense variants in clinical settings (41). Analysis of the cardiac arrhythmia panel variants identified 12 of 26 (46%) predicted to be pathogenic by at least three models (i.e., Damaging, Probably damaging, Possibly damaging, Deleterious, or Disease causing). Analysis of the epilepsy panel variants identified 20 of 31 (65%) predicted to be pathogenic by at least three models.

**Figure 1:**
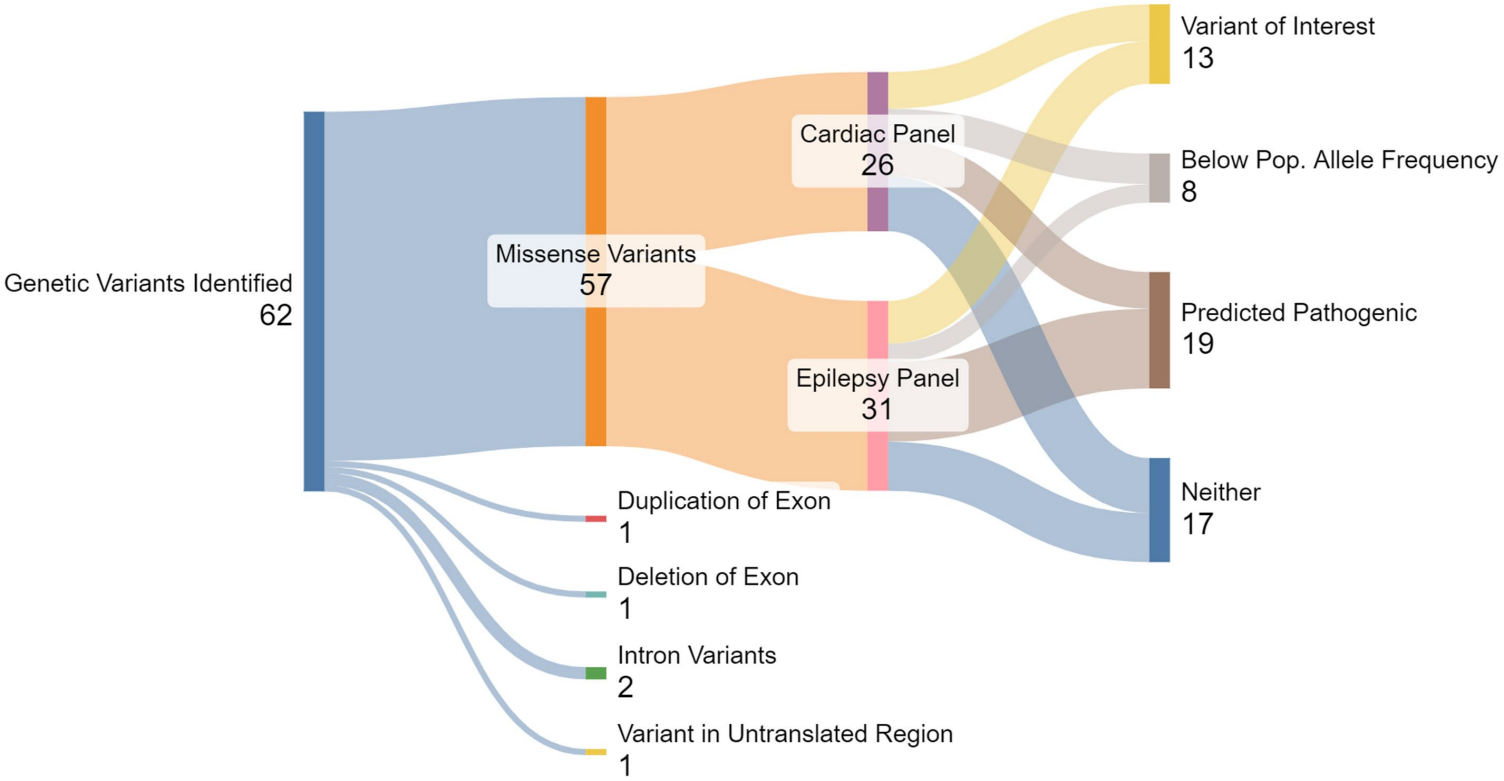
Summary of gene testing results. From the total 62 genetic variants detected, 57 were missense variants, comprised of 27 genes from the cardiac panel and 30 from the epilepsy panel. Multiple *in silico* algorithms predicted that a total of 32 variants were pathogenic, and a total of 22 variants were below the pre-determined population allele frequency threshold (<0.00004). Thirteen variants, seven from the cardiac panel and six from the epilepsy panel, were both predicted pathogenic and below population allele frequency threshold, identifying them as SUDEP Variants of Interest (VoI).

A population allele frequency of any gene that was less than our calculated threshold (<0.00004), suggests they are less frequent than expected if the variants were benign. We identified 11 of 26 (42%) cardiac variants and 10 of 31 (32%) epilepsy variants with population allele frequencies below threshold. Of these variants, six cardiac variants and seven epilepsy variants were also predicted to be pathogenic by at least three *in silico* models. These 13 variants, identified across 11 individuals, were categorized as SUDEP variants of interest (VoI; Figure 1). Phenotypes commonly associated with each of the genes included among the 13 VoI are presented in Table 5; Protein expression profiles are provided in Supplemental Table S2.

**Table 5:** Epilepsy and cardiac phenotypes commonly associated with the genes identified as Variants of Interest.

Gene	Phenotype(s)
Epilepsy genes	
*CACNA1A*	DEE
*CDKL5*	DEE
*EPM2A*	Lafora disease
*GRIN2B*	DEE
*MFSD8*	NCL
*SCN1A*	DEE (Dravet syndrome), febrile seizures, GEFS+, myoclonic atonic epilepsy
*SCN2A*	DEE, GEFS+, Self-limited familial and non-familial infantile epilepsy
Cardiac genes	
*DSG2*	ARVC, DCM
*KCNE1*	LQTS, JLNS
*RYR2*	ARVC, CPVT, LQTS, DCM, SCD, SUDS
*SCN5A*	ARVC, BrS, DCM, HB, LQTS, SIDS, SSS
*SCN10A*	BrS, LQTS, AF, painful small-fiber peripheral neuropathy

Abbreviations: AF – Atrial fibrillation; ARVC – Arrhythmogenic right ventricular cardiomyopathy; BrS – Brugada syndrome; CPVT – Catecholaminergic polymorphic ventricular tachycardia; DCM – Dilated cardiomyopathy; DEE – Developmental and epileptic encephalopathy; GEFS+ - Generalized epilepsy with febrile seizures plus; HB – Heart block; JLNS – Jervell and Lange-Nielsen syndrome; LQTS – Long QT syndrome; NCL – Neuronal Ceroid Lipofuscinosis; SCD – Sudden cardiac death; SIDS – Sudden infant death syndrome; SSS – Sick sinus syndrome; SUDS – Sudden unexpected death syndrome.

We compared demographic and clinical factors between decedents with SUDEP VoI to everyone else. Age, sex, seizure frequency, and number of ASM were not significantly different between those with VoI (*n* = 14) and those without (*n* = 25) (Age: W = 159, *p* = 0.89; Sex: Fisher’s exact test *p* = 0.14; Seizure frequency: Fisher’s exact test *p* = 0.60; ASM: Fisher’s exact test *p* = 0.88).

This cohort contained novel variants with no corresponding ClinVar entry. From the cardiac panel, two variants were identified – *SCN10A* c.5339C > T (p.Pro1780Leu), and *SCN5A* c.5286C > G, (p.Ile1762Met); Both were classified as SUDEP genes of interest. From the epilepsy panel one novel variant was identified – *GRIN2B* c.3994G > T (p.Asp132Tyr). The *GRIN2B* variant met our criteria for a SUDEP VoI.

An *SCN10A* variant c.3803G > A (p.Arg1268Gln) was the only identical variant documented in multiple SUDEP cases in our cohort (*n* = 3). Two additional *SCN10A* variants were identified in the cohort, making *SCN10A* the most frequent gene finding. Multiple variants were also reported in more than one decedent for *RYR2* (*n* = 4), as well as *DSG2*, *LMNA*, *PIGA*, *PIGO*, *PNKP*, *SCN5A*, *SNTA1*, and *TTN* variants (*n* = 2). All other genes were observed in single cases.

## Discussion

More than half of the decedents in this SUDEP cohort (21/39; 54%) had genetic variants in both epilepsy and cardiac gene panels. Over half of the missense variants identified on either panel were predicted to be pathogenic by three or more *in silico* algorithms, and over a third of the variants were compatible with pathogenicity based on population allele frequency (46). Together, these metrics identified 13 SUDEP Variants of Interest (VoI), 23% of all missense variants in this cohort. Our results contribute to a growing literature on the possible contribution of cardiac genetic variants to epilepsy mortality, and SUDEP, specifically.

The circumstances surrounding death in this cohort were typical of SUDEP characteristics; death was most frequently reported during the night, the deceased were found in bed, death was unwitnessed, and there was evidence of a preceding seizure. Interestingly, many of the typical features of sudden cardiac death are similar to those of SUDEP, including occurring during the night, the deceased is found in bed in a prone position, the death is unwitnessed, and post-mortem investigation fails to identify a cause of death (49–51).

It is well-established that epilepsy negatively affects heart health, independent of any present gene variants. Epidemiological studies have found increased rates of heart disease and sudden cardiac death in adults diagnosed with epilepsy compared to the general population (21). Post-mortem findings of PwE have identified myocardial structural changes such as fibrosis, myocyte vacuolization (52–54). These findings have led to the hypothesis that surges in catecholamines and hypoxemia due to repeated seizures lead to electrical and mechanical heart dysfunction (22, 55–58). In addition, while no single ASM, nor the presence of polytherapy or monotherapy have been reliably associated with SUDEP (59, 60), it should be noted that some ASM have been shown to modulate cardiac activity. For example, therapeutic doses of carbamazepine may induce bradyarrhythmia in some patients (61, 62) and both ASM use and sudden withdrawal of ASM have been shown to reduce heart-rate variability (63, 64) which has been associated with an increase in sudden cardiac death (65). These effects could be relevant in genetically susceptible individuals. Two of the most common ASMs reported in this cohort included phenytoin and carbamazepine, which in the setting of certain cardiac conditions (e.g., Brugada syndrome) should be avoided. Notably, at least three decedents in this cohort with either *SCN5A* or *SCN10A* variants reported using one of these two ASMs (cases: 03, 04, 32).

In part because of these heart-brain interactions, proving a cardiac genetic contribution to SUDEP is difficult. If such a relationship exists, a cardiac-related genetic contribution to SUDEP could take multiple forms: (1) a subclinical arrhythmogenic variant that increases the susceptibility to SUDEP (66); (2) a genetic variant that confers both a risk of epilepsy and a risk of sudden cardiac death (i.e., monogenic/arrhythmogenic epilepsy) (66); (3) the co-occurrence of different epilepsy-causing and arrhythmia-causing genes that independently increase the risk for sudden death (i.e., polygenic) (25). Polygenic risk for SUDEP may overlap with polygenic risk for sudden cardiac death and the proportion of SUDEP deaths that may be poly- or oligemic compared to monogenic is not known.

### Sudden unexpected death in epilepsy variants of interest

At least three *in silico* algorithms classified 13 variants in the cardiac panel and 19 variants in the epilepsy panel as pathogenic, totaling 32 (56%). Based on population allele frequency, 12 variants in the cardiac panel and nine in the epilepsy panel were less than the threshold 0.00004, totaling 21 (37%). Together, these metrics identified a total of 13 (23%) of the 57 missense variants in this cohort as SUDEP VoI. The 13 VoI were identified in 11 decedents (two individuals had two: cases 03 and 46).

In both epilepsy and cardiac gene panels, ion channels were the most common protein types encoded by the *in silico-*predicted pathogenic genes, including sodium, calcium, potassium, and chloride channels. Other groups of proteins identified in this cohort included cell adhesion proteins & ion channel regulators, protein & solute transports, cell signaling molecules, cell replication & DNA repair proteins, and proteins regulating metabolism.

Among the 13 SUDEP VoI, nine of the genes encode ion channel subunits (five of the nine were from the cardiac panel), one encodes a cell signaling molecule (epilepsy panel), one encodes a cell adhesion protein (cardiac panel), one phosphatase (epilepsy panel), and one a protein/solute transport (epilepsy panel). Most of the genes with identified VoI are autosomal dominant inheritance, excepting *MFSD8* (recessive), *EPM2A* (recessive), and *CDKL5* (x-linked).

The exact mechanisms of disease are not known for some of the cardiac phenotypes associated with the genes in this panel. However, there are many ways that proteins linked to cardiac phenotypes could contribute to SUDEP. Ion channel dysfunction provides perhaps the most straightforward explanation of sudden death. Ion channels are critical to the formation and propagation of action potentials, both in the heart and brain. Dysregulation of these time- and voltage-dependent mechanisms could lead to heart failure directly or cardiorespiratory arrest via spreading depolarization in the autonomic nervous system – all proposed mechanisms of SUDEP (15, 67, 68). Proteins involved in cell adhesion (e.g., *DSG2*) or protein transport (e.g., *MFSD8*) could alter the structural architecture of the heart which could lead directly to a cardiac phenotype or could reduce resistance to mechanical stress which may be experienced during a seizure (69, 70). Regardless of the mechanism, a variant in an arrhythmogenic- or cardiomyopathy-related gene could predispose a person with epilepsy to sudden death by exacerbating the impacts of recurrent seizures on heart health or the effect of a single prolonged convulsive seizure.

### Relation to previous work

A recent systematic review of the genetics of SUDEP concluded that the most prominent and frequent gene variants identified across studies were associated with ion channels – which may be co-expressed in the heart and brain and are causally related to some epilepsies (e.g., DS and *SCN1A*) and cardiac disorders (e.g., Brugada Syndrome and *SCN5A*) – and genes linked to cardiac arrhythmia phenotypes (25). However, most of the genes identified had VUS ratings by *in silico* pathogenicity models. Importantly, although protein dysfunction in these genes may lead to disease (channelopathies) associated with the heart or the brain, almost all these ion channels are expressed to some degree in both the heart and the brain (Supplemental Table S2), opening the possibility of a shared genetic susceptibility between epilepsy and cardiac arrhythmias.

Previous post-mortem investigations of SUDEP cohorts have reported many of the same genes identified in this SUDEP cohort. These include the epilepsy genes *CACNA1A*, *CDKL5*, *CHRNA4*, *GRIN2B*, *NRXN1*, *PNKP*, *POLG*, *SCARB2*, *SCN1A*, and *SCN2A* as well as the cardiac genes *ANK2*, *KCNE1*, *KCNQ1*, *RYR2*, *SCN5A*, and *SCN10A* (26, 30, 31, 33, 34, 71, 72). Many of these genes, from both panels, were reported across multiple studies, including the ion channel subunits for sodium (*SCNs*), potassium (*KCNs*), and calcium (*CACNA1A*, *RYR2*). These genes constitute a high priority for future investigations of genetic biomarkers for SUDEP.

### Future work

More than half of our cohort had variants identified in the comprehensive cardiac arrhythmia panel, however, the role these genetic variants played in sudden death is unclear. Further work to determine the significance of the identified variants is needed. Ultimately, genetic risk factors could be combined with other clinical risk factors to develop individualized SUDEP risk prediction tools.

Concerted efforts are needed to investigate the contribution of cardiac genotypes to epilepsy mortality. Few of the genes identified in our cohort, as well as in the broader cardiac SUDEP literature, have had functional studies. Among the epilepsy panel findings in our cohort, we were only able to find functional studies for the two known disease-associated variants in the genes *EPM2A* (73) and *SCN1A* (74). Among the cardiac panel findings, we found functional studies for five variants in the genes *ANK2* (75, 76), *DSG2* (77), *KCNQ1* (78), *LMNA* (79, 80), and *SCN10A* (81). Evaluation by *in vivo* and *in vitro* methods to assess potential pathogenic mechanisms are critical steps in validating the remainder of these gene variants as SUDEP biomarkers, particularly those found in multiple SUDEP cohorts.

Cardiac contributions to epilepsy mortality are well-recognized and beginning to receive appropriate research attention. However, SUDEP mechanisms are likely heterogeneous and only a subset is likely attributable to cardiac dysfunction. The MORTEMUS study, which systematically reviewed cardiorespiratory events captured in epilepsy monitoring units, showed that primary cardiac arrhythmia is rarely an isolated primary cause of SUDEP in a cohort of people admitted to epilepsy monitoring units for evaluation (12). Thus, genetic screening should be expanded to other potential mechanisms, such as respiratory disorders (33, 82, 83), the serotonergic system (67, 84, 85), and the immune system (86–88).

## Limitations

This cohort was identified through a retrospective case series review. At the time of this study, GeneDx comprehensive epilepsy and cardiac panels included 87 and 46 genes, respectively. Currently, these panels include 144 and 58 genes, respectively. As is common in genetics case series, variants were assessed for pathogenicity through multiple *in silico* algorithms. Our study was the first to apply a population allele frequency threshold to filter variants of interest. Although the method used here was novel in the context of SUDEP genetics, the variants of interest we identified did not meet the definite criteria for pathogenicity based on the guidelines published by the ACMG/AMP (41) and there is not substantial evidence that these genes are causative of SUDEP. The majority of the gene variants identified in this cohort were classified as VUS. This classification pertains to their relationship to cardiac or epilepsy phenotypes, not SUDEP. However, they may still be relevant to SUDEP in some contexts. Particularly, the variants in the cardiac genes may be impactful in people with epilepsy, even if they produce subclinical phenotypes in otherwise healthy individuals. These variants may be worth further investigation; variants can be reclassified over time as more evidence becomes available.

Access to clinical data was limited, including epilepsy etiology, seizure semiology, and cardiac medical histories. Although autopsy reports did not identify a cause of death that would rule out SUDEP, we cannot be certain all relevant comorbidities were reported for all cases. Genetic testing of family members was not available, which could have provided clinically useful evidence for the relationship between cardiac arrhythmias and epilepsy. Based on our findings and the results of previous reports, genetic testing should be performed in first degree relatives after sudden death. SUDEP within a family with a common gene variant would be among the strongest evidence for a genetic influence on SUDEP. Moreover, it would allow for appropriate screening and management of relatives for those who genetic variants are identified. Few reports of multiple cases of SUDEP within a family have been published (89–91).

A persistent limitation across studies of SUDEP genetics is the lack of standardization in methods, making comparison difficult. First, populations and ascertainment methods differed, with a mix of prospective and retrospective sampling, with few reaching the level of population-based sampling. Second, different genes were assessed, and the method of genetic testing varied. Some studies used whole genome or whole exome sequencing, while others were limited to genetic panels provided by various services. Finally, the criteria for determining pathogenicity differed; most studies use some combination of *in silico* algorithms and some also compared to living epilepsy groups. Importantly, all studies examined at an outcome (SUDEP) within a pathogenic population (people with epilepsy), so it is possible that these variants may confer risk of epilepsy and be non-contributory to the SUD.

## Conclusion

Targeted epilepsy and cardiac arrhythmia gene panels detected variants in 28 of 39 (72%) SUDEP cases. Over half of this cohort had epilepsy variants (54%) and over half had cardiac variants (54%), with more than a third having variants in both panels (36%). Nearly two-thirds of these variants were predicted to be pathogenic by at least three of four *in silico* algorithms, while one-third of the cardiac variants and one-fifth of the epilepsy variants had population allele frequencies below threshold. These results identified 13 priority targets for future functional studies of these genes potential role in sudden death.

The proportion of cases with cardiac variants aligns with recent studies of the disproportionate cardiac burden the epilepsy community faces compared to the general population and suggests a possible cardiac contribution to SUDEP. This data also supports the concept of a collaborative care approach between neurology and cardiology, as well as pathology and genetics disciplines. Standard cardiac screening such as 12-lead ECG or Holter monitor could be increasingly utilized in epilepsy care at little cost. Targeting these initiatives to individuals with channelopathies may be particularly effective, given their well-recognized role in severe epilepsies and cardiac arrhythmias.

This data demonstrates the necessity for further exploration of potential genetic contributions to SUDEP. Such endeavors will (1) allow for identification of high-risk individuals which can aid in enriched cohorts for clinical trials and (2) allow for design of targeted therapies to prevent SUDEP. Valuable information would be gained through protocols that identify genetic predispositions in individuals with epilepsy, and that additional value may be obtained from extending genetic screening to parents, siblings, and children after the sudden death of an individual with epilepsy. Epilepsy lags other disease fields, including cardiac arrhythmias, in incorporating genetic analyses as part of pathology analysis. Improvements in technology have made genetic testing fast and cost-effective, and uptake in the use of genetic analyses aligns with precision/individualized medicine goals adopted by many health care institutions. Widespread uptake of genetic screening in PwE could facilitate research efforts to identify SUDEP biomarkers.

## Data availability statement

The original contributions presented in the study are included in the article/Supplementary material, further inquiries can be directed to the corresponding author/s.

## Ethics statement

The studies involving humans were approved by The Hospital for Sick Children Research Ethics Board. The studies were conducted in accordance with the local legislation and institutional requirements. The human samples used in this study were acquired from the Ontario Forensic Pathology Service (OFPS) which is tasked with investigation of all sudden or unexpected deaths in Ontario, Canada. Written informed consent for participation was not required from the participants or the participants’ legal guardians/next of kin in accordance with the national legislation and institutional requirements.

## Author contributions

AA: Writing – review & editing, Writing – original draft, Visualization, Methodology, Investigation, Formal analysis, Data curation. AK: Writing – review & editing, Methodology, Conceptualization. AW: Writing – review & editing, Resources, Methodology, Investigation, Data curation. RW: Writing – review & editing, Visualization, Validation. KC: Writing – review & editing, Methodology, Data curation, Conceptualization. RH: Writing – review & editing. MP: Writing – review & editing, Resources, Methodology, Investigation, Data curation, Conceptualization. ED: Writing – review & editing, Supervision, Resources, Methodology, Funding acquisition, Data curation, Conceptualization.

## Footnotes

^1^ https://braininstitute.ca/epilepsy-psp

^2^ https://www.ncbi.nlm.nih.gov/clinvar/

^3^ http://sift.jcvi.org/

^4^ https://www.jcvi.org/research/provean

^5^ http://genetics.bwh.harvard.edu/pph2/

^6^ https://www.mutationtaster.org/

^7^ https://gnomad.broadinstitute.org/

## References

[1] Forsgren L Hauser WA Olafsson E Sander JWA Sillanpää M Tomson T. Mortality of epilepsy in developed countries: a review. Epilepsia. (2005) 46:18–27. doi: 10.1111/j.1528-1167.2005.00403.x 16393174

[2] Ficker DM So EL Shen WK Annegers JF O’Brien PC Cascino GD. Population-based study of the incidence of sudden unexplained death in epilepsy. Neurology. (1998) 51:1270–4. doi: 10.1212/WNL.51.5.1270, PMID: 9818844

[3] Walczak TS Leppik IE D’Amelio M Rarick J So E Ahman P. Incidence and risk factors in sudden unexpected death in epilepsy: a prospective cohort study. Neurology. (2001) 56:519–25. doi: 10.1212/WNL.56.4.519 11222798

[4] Sveinsson O Andersson T Carlsson S Tomson T. The incidence of SUDEP: a nationwide population-based cohort study. Neurology. (2017) 89:170–7. doi: 10.1212/WNL.0000000000004094 28592455

[5] Keller AE Whitney R Li SA Pollanen MS Donner EJ. Incidence of sudden unexpected death in epilepsy in children is similar to adults. Neurology. (2018) 91:e107–11. doi: 10.1212/WNL.0000000000005762, PMID: 29884734

[6] Nashef L So EL Ryvlin P Tomson T. Unifying the definitions of sudden unexpected death in epilepsy. Epilepsia. (2012) 53:227–33. doi: 10.1111/j.1528-1167.2011.03358.x, PMID: 22191982

[7] Cooper MS McIntosh A Crompton DE McMahon JM Schneider A Farrell K. Mortality in Dravet syndrome. Epilepsy Res. (2016) 128:43–7. doi: 10.1016/j.eplepsyres.2016.10.006 27810515

[8] Shmuely S Sisodiya SM Gunning WB Sander JW Thijs RD. Mortality in Dravet syndrome: a review. Epilepsy Behav. (2016) 64:69–74. doi: 10.1016/j.yebeh.2016.09.007 27732919

[9] Kelley MS Jacobs MP Lowenstein DH. The NINDS epilepsy research benchmarks. Epilepsia. (2009) 50:579–82. doi: 10.1111/j.1528-1167.2008.01813.x 19317887 PMC2874963

[10] DeGiorgio CM Curtis A Hertling D Moseley BD. Sudden unexpected death in epilepsy: Risk factors, biomarkers, and prevention. Acta Neurol Scand. (2019) 139:220–30. doi: 10.1111/ane.13049 30443951

[11] Szurhaj W Leclancher A Nica A Périn B Derambure P Convers P. Cardiac autonomic dysfunction and risk of sudden unexpected death in epilepsy. Neurology. (2021) 96:998. doi: 10.1212/WNL.0000000000011998 33837114

[12] Ryvlin P Nashef L Lhatoo SD Bateman LM Bird J Bleasel A. Incidence and mechanisms of cardiorespiratory arrests in epilepsy monitoring units (MORTEMUS): a retrospective study. Lancet Neurol. (2013) 12:966–77. doi: 10.1016/S1474-4422(13)70214-X 24012372

[13] Lhatoo SD Nei M Raghavan M Sperling M Zonjy B Lacuey N. Nonseizure SUDEP: sudden unexpected death in epilepsy without preceding epileptic seizures. Epilepsia. (2016) 57:1161–8. doi: 10.1111/epi.13419, PMID: 27221596 PMC5541994

[14] Barot N Nei M. Autonomic aspects of sudden unexpected death in epilepsy (SUDEP). Clin Auton Res. (2019) 29:151–60. doi: 10.1007/s10286-018-0576-1 30456432

[15] Nashef L Hindocha N Makoff A. Risk factors in sudden death in epilepsy (SUDEP): the quest for mechanisms. Epilepsia. (2007) 48:859–71. doi: 10.1111/j.1528-1167.2007.01082.x 17433051

[16] Hughes JR. A review of sudden unexpected death in epilepsy: prediction of patients at risk. Epilepsy Behav. (2009) 14:280–7. doi: 10.1016/j.yebeh.2008.12.004, PMID: 19130900

[17] Sveinsson O Andersson T Carlsson S Tomson T. Circumstances of SUDEP: a nationwide population-based case series. Epilepsia. (2018) 59:1074–82. doi: 10.1111/epi.14079, PMID: 29663344

[18] Sveinsson O Andersson T Mattsson P Carlsson S Tomson T. Clinical risk factors in SUDEP: a nationwide population-based case-control study. Neurology. (2020) 94:e419–29. doi: 10.1212/WNL.0000000000008741, PMID: 31831600 PMC7079690

[19] Ryvlin P Cucherat M Rheims S. Risk of sudden unexpected death in epilepsy in patients given adjunctive antiepileptic treatment for refractory seizures: a meta-analysis of placebo-controlled randomised trials. Lancet Neurol. (2011) 10:961–8. doi: 10.1016/S1474-4422(11)70193-4, PMID: 21937278

[20] Harden C Tomson T Gloss D Buchhalter J Cross JH Donner E. Practice guideline summary: sudden unexpected death in epilepsy incidence rates and risk factors: report of the guideline development, dissemination, and implementation subcommittee of the American Academy of Neurology and the American Epilepsy Society. Epilepsy Curr. (2017) 17:180–7. doi: 10.5698/1535-7511.17.3.180, PMID: 28684957 PMC5486432

[21] Costagliola G Orsini A Coll M Brugada R Parisi P Striano P. The brain-heart interaction in epilepsy: implications for diagnosis, therapy, and SUDEP prevention. Ann Clin Transl Neurol. (2021) 8:1557–68. doi: 10.1002/acn3.51382 34047488 PMC8283165

[22] Verrier RL Pang TD Nearing BD Schachter SC. The epileptic heart: concept and clinical evidence. Epilepsy Behav. (2020) 105:106946. doi: 10.1016/j.yebeh.2020.106946 32109857

[23] Gould L Reid C-A Rodriguez AJ Devinsky O For SUDC Video Working Group. Video analyses of sudden unexplained deaths in toddlers. Neurology. (2024) 102:e208038. doi: 10.1212/WNL.0000000000208038 38175965 PMC11097764

[24] Devinsky O Friedman D Cheng JY Moffatt E Kim A Tseng ZH. Underestimation of sudden deaths among patients with seizures and epilepsy. Neurology. (2017) 89:886–92. doi: 10.1212/WNL.0000000000004292, PMID: 28768851 PMC5577966

[25] Chahal CAA Salloum MN Alahdab F Gottwald JA Tester DJ Anwer LA. Systematic review of the genetics of sudden unexpected death in epilepsy: potential overlap with sudden cardiac death and arrhythmia-related genes. J Am Heart Assoc. (2020) 9:e012264. doi: 10.1161/JAHA.119.012264, PMID: 31865891 PMC6988156

[26] Friedman D Kannan K Faustin A Shroff S Thomas C Heguy A. Cardiac arrhythmia and neuroexcitability gene variants in resected brain tissue from patients with sudden unexpected death in epilepsy (SUDEP). NPJ Genom Med. (2018) 3:9. doi: 10.1038/s41525-018-0048-5 29619247 PMC5869741

[27] Verducci C Friedman D Donner E Devinsky O. Genetic generalized and focal epilepsy prevalence in the north American SUDEP registry. Neurology. (2020) 94:e1757–63. doi: 10.1212/WNL.0000000000009295 32217773 PMC7282874

[28] Glasscock E. Genomic biomarkers of SUDEP in brain and heart. Epilepsy Behav. (2014) 38:172–9. doi: 10.1016/j.yebeh.2013.09.019, PMID: 24139807 PMC3989471

[29] Bardai A Blom MT Van Noord C Verhamme KM Sturkenboom MCJM Tan HL. Sudden cardiac death is associated both with epilepsy and with use of antiepileptic medications. Heart. (2015) 101:17–22. doi: 10.1136/heartjnl-2014-305664 25031263

[30] Tu E Bagnall RD Duflou J Semsarian C. Post-mortem review and genetic analysis of sudden unexpected death in epilepsy (SUDEP) cases. Brain Pathol. (2011) 21:201–8. doi: 10.1111/j.1750-3639.2010.00438.x, PMID: 20875080 PMC8094243

[31] Coll M Allegue C Partemi S Mates J Del Olmo B Campuzano O. Genetic investigation of sudden unexpected death in epilepsy cohort by panel target resequencing. Int J Legal Med. (2016) 130:331–9. doi: 10.1007/s00414-015-1269-0 26423924

[32] Partemi S Vidal MC Striano P Campuzano O Allegue C Pezzella M. Genetic and forensic implications in epilepsy and cardiac arrhythmias: a case series. Int J Legal Med. (2015) 129:495–504. doi: 10.1007/s00414-014-1063-4, PMID: 25119684

[33] Bagnall RD Crompton DE Petrovski S Lam L Cutmore C Garry SI. Exome-based analysis of cardiac arrhythmia, respiratory control, and epilepsy genes in sudden unexpected death in epilepsy. Ann Neurol. (2016) 79:522–34. doi: 10.1002/ana.24596, PMID: 26704558

[34] Ge Y Ding D Zhu G Kwan P Wang W Hong Z. Genetic variants in incident SUDEP cases from a community-based prospective cohort with epilepsy. J Neurol Neurosurg Psychiatry. (2019) 91:126–31. doi: 10.1136/jnnp-2019-321983 31776209

[35] Auerbach DS Jones J Clawson BC Offord J Lenk GM Ogiwara I. Altered cardiac electrophysiology and SUDEP in a model of Dravet syndrome. PLoS One. (2013) 8:e77843. doi: 10.1371/journal.pone.0077843, PMID: 24155976 PMC3796479

[36] Kalume F Westenbroek RE Cheah CS Yu FH Oakley JC Scheuer T. Sudden unexpected death in a mouse model of Dravet syndrome. J Clin Invest. (2013) 123:1798–808. doi: 10.1172/JCI66220, PMID: 23524966 PMC3613924

[37] Frasier CR Wagnon JL Bao YO McVeigh LG Lopez-Santiago LF Meisler MH. Cardiac arrhythmia in a mouse model of sodium channel SCN8A epileptic encephalopathy. Proc Natl Acad Sci USA. (2016) 113:12838–43. doi: 10.1073/pnas.1612746113, PMID: 27791149 PMC5111690

[38] Goldman AM Glasscock E Yoo J Chen TT Klassen TL Noebels JL. Arrhythmia in heart and brain: KCNQ1 mutations link epilepsy and sudden unexplained. Sci Transl Med. (2009) 1:2ra6. doi: 10.1126/scitranslmed.3000289, PMID: 20368164 PMC2951754

[39] Lehnart SE Mongillo M Bellinger A Lindegger N Chen B-X Hsueh W. Leaky ca 2+ release channel/ryanodine receptor 2 causes seizures and sudden cardiac death in mice. J Clin Invest. (2008) 118:346. doi: 10.1172/JCI35346 PMC2381750 18483626

[40] Aiba I Wehrens XHT Noebels JL. Leaky RyR2 channels unleash a brainstem spreading depolarization mechanism of sudden cardiac death. Proc Natl Acad Sci USA. (2016) 113:E4895–903. doi: 10.1073/pnas.1605216113, PMID: 27482086 PMC4995941

[41] Richards S Aziz N Bale S Bick D Das S Gastier-Foster J. Standards and guidelines for the interpretation of sequence variants: a joint consensus recommendation of the American College of Medical Genetics and Genomics and the Association for Molecular Pathology. Genet Med. (2015) 17:405–24. doi: 10.1038/gim.2015.30, PMID: 25741868 PMC4544753

[42] Ng PC Henikoff S. Predicting deleterious amino acid substitutions. Genome Res. (2001) 11:863–74. doi: 10.1101/gr.176601 11337480 PMC311071

[43] Choi Y Sims GE Murphy S Miller JR Chan AP. Predicting the functional effect of amino acid substitutions and indels. PLoS One. (2012) 7:688. doi: 10.1371/journal.pone.0046688 PMC3466303 23056405

[44] Adzhubei IA Schmidt S Peshkin L Ramensky VE Gerasimova A Bork P. A method and server for predicting damaging missense mutations. Nat Methods. (2010) 7:248–9. doi: 10.1038/nmeth0410-248, PMID: 20354512 PMC2855889

[45] Steinhuas R Proft S Schuelke M Cooper DN Schwarz JM Seelow D. MutationTaster2021. Nucleic Acids Res. (2021) 49:W446–51. doi: 10.1093/nar/gkab266, PMID: 33893808 PMC8262698

[46] Whiffin N Minikel E Walsh R O’Donnell-Luria AH Karczewski K Ing AY. Using high-resolution variant frequencies to empower clinical genome interpretation. Genet Med. (2017) 19:1151–8. doi: 10.1038/gim.2017.26, PMID: 28518168 PMC5563454

[47] Lhatoo SD Solomon JK McEvoy AW Kitchen ND Shorvon SD Sander JW. A prospective study of the requirement for and the provision of epilepsy surgery in the United Kingdom. Epilepsia. (2003) 44:673–6. doi: 10.1046/j.1528-1157.2003.46002.x, PMID: 12752466

[48] Keller AE Ho J Whitney R Li SA Williams AS Pollanen MS. Autopsy-reported cause of death in a population-based cohort of sudden unexpected death in epilepsy. Epilepsia. (2021) 62:472–80. doi: 10.1111/epi.16793, PMID: 33400291

[49] Ryvlin P Cascino GD. Sudden unexpected death in epilepsy patients is often misdiagnosed as sudden cardiac death. Neurol. Genet. (2017) 89:878–9. doi: 10.1212/WNL.0000000000004301 28768845

[50] Dasheiff RM. Sudden unexpected death in epilepsy: a series from an epilepsy surgery program and speculation on the relationship to sudden cardiac death. J Clin Neurophysiol. (1991) 8:216–22. doi: 10.1097/00004691-199104000-00010 2050822

[51] Gami AS Olson EJ Shen WK Wright RS Ballman KV Hodge DO. Obstructive sleep apnea and the risk of sudden cardiac death: a longitudinal study of 10,701 adults. J Am Coll Cardiol. (2013) 62:610–6. doi: 10.1016/j.jacc.2013.04.080, PMID: 23770166 PMC3851022

[52] Falconer B Rajs J. Post-mortem findings of cardiac lesions in epileptics: a preliminary report. Forensic Sci. (1976) 8:63–71. doi: 10.1016/0300-9432(76)90048-0 824190

[53] Leestma JE Walczak T Hughes JR Kalelkar MB Teas SS. A prospective study on sudden unexpected death in epilepsy. Ann Neurol. (1989) 26:195–203. doi: 10.1002/ana.410260203 2774506

[54] Natelson BH Suarez RV Terrence CF Turizo R. Patients with epilepsy who die suddenly have cardiac disease. Arch Neurol. (1998) 55:857–60. doi: 10.1001/archneur.55.6.857, PMID: 9626779

[55] Blum AS Ives JR Goldberger AL Al-Aweel IC Krishnamurthy KB Drislane FW. Oxygen desaturations triggered by partial seizures: implications for cardiopulmonary instability in epilepsy. Epilepsia. (2000) 41:536–41. doi: 10.1111/j.1528-1157.2000.tb00206.x, PMID: 10802758

[56] Fineschi V Silver MD Karch SB Parolini M Turillazzi E Pomara C. Myocardial disarray: an architectural disorganization linked with adrenergic stress? Int J Cardiol. (2005) 99:277–82. doi: 10.1016/j.ijcard.2004.01.022, PMID: 15749187

[57] Moseley B Bateman L Millichap JJ Wirrell E Panayiotopoulos CP. Autonomic epileptic seizures, autonomic effects of seizures, and SUDEP. Epilepsy Behav. (2013) 26:375–85. doi: 10.1016/j.yebeh.2012.08.020 23099286

[58] Nass RD Motloch LJ Paar V Lichtenauer M Baumann J Zur B. Blood markers of cardiac stress after generalized convulsive seizures. Epilepsia. (2019) 60:201–10. doi: 10.1111/epi.14637, PMID: 30645779

[59] Hesdorffer DC Tomson T. Sudden unexpected death in epilepsy: potential role of antiepileptic drugs. CNS Drugs. (2013) 27:113–9. doi: 10.1007/s40263-012-0006-1, PMID: 23109241

[60] Hesdorffer DC Tomson T Benn E Sander JW Nilsson L Langan Y. Do antiepileptic drugs or generalized tonic-clonic seizure frequency increase SUDEP risk? A combined analysis. Epilepsia. (2012) 53:249–52. doi: 10.1111/j.1528-1167.2011.03354.x 22191685

[61] Kasarskis EJ Kuo CS Berger R Nelson KR. Carbamazepine-lnduced cardiac dysfunction: characterization of two distinct clinical syndromes. Arch Intern Med. (1992) 152:186–91. doi: 10.1001/archinte.1992.00400130184025 1728915

[62] Kenneback G Bergfeldt L Tomson T Spina E Edhag O. Carbamazepine induced bradycardia - a problem in general or only in susceptible patients? A 24-h long-term electrocardiogram study. Epilepsy Res. (1992) 13:141–5. doi: 10.1016/0920-1211(92)90069-6 1464298

[63] Kennebäck G Ericson M Tomson T Bergfeldt L. Changes in arrhythmia profile and heart rate variability during abrupt withdrawal of antiepileptic drugs. Seizure. (1997) 6:369–75. doi: 10.1016/S1059-1311(97)80036-2, PMID: 9663800

[64] Lotufo PA Valiengo L Bensenor IM Brunoni AR. A systematic review and meta-analysis of heart rate variability in epilepsy and antiepileptic drugs. Epilepsia. (2012) 53:272–82. doi: 10.1111/j.1528-1167.2011.03361.x, PMID: 22221253

[65] Bigger JT Fleiss JL Steinman RC Rolnitzky LM Kleiger RE Rottman JN. Frequency domain measures of heart period variability and mortality after myocardial infarction. Circulation. (1992) 85:164–71. doi: 10.1161/01.CIR.85.1.164, PMID: 1728446

[66] Bleakley LE Soh MS Bagnall RD Sadleir LG Gooley S Semsarian C. Are variants causing cardiac arrhythmia risk factors in sudden unexpected death in epilepsy? Front Neurol. (2020) 11:925. doi: 10.3389/fneur.2020.00925, PMID: 33013630 PMC7505992

[67] Richerson GB Buchanan GF. The serotonin axis: shared mechanisms in seizures, depression, and SUDEP. Epilepsia. (2011) 52:28–38. doi: 10.1111/j.1528-1167.2010.02908.x, PMID: 21214537 PMC3052632

[68] Aiba I Noebels JL. Spreading depolarization in the brainstem mediates sudden cardiorespiratory arrest in mouse SUDEP models. Sci Transl Med. (2015) 7:50. doi: 10.1126/scitranslmed.aaa4050 PMC4852131 25855492

[69] Kadam SD Sullivan BJ Goyal A Blue ME Smith-Hicks C. Rett syndrome and CDKL5 deficiency disorder: from bench to clinic. Int J Mol Sci. (2019) 20:5098. doi: 10.3390/ijms20205098, PMID: 31618813 PMC6834180

[70] Crasto S My I Di Pasquale E. The broad spectrum of LMNA cardiac diseases: from molecular mechanisms to clinical phenotype. Front Physiol. (2020) 11:761. doi: 10.3389/fphys.2020.00761, PMID: 32719615 PMC7349320

[71] Coll M Striano P Ferrer-Costa C Campuzano O Mates J del Olmo B. Targeted next-generation sequencing provides novel clues for associated epilepsy and cardiac conduction disorder/SUDEP. PLoS One. (2017) 12:e0189618. doi: 10.1371/journal.pone.0189618, PMID: 29261713 PMC5736193

[72] Hata Y Yoshida K Kinoshita K Nishida N. Epilepsy-related sudden unexpected death: targeted molecular analysis of inherited heart disease genes using next-generation DNA sequencing. Brain Pathol. (2017) 27:292–304. doi: 10.1111/bpa.12390 27135274 PMC8028934

[73] García-Gimeno MA Viana R Rubio-Villena C Sánchez-Martín P Brewer MK Gentry MS.. *Phenotypic characterization of a new EPM2A mutation (N163D)*. 2nd biennial international Lafora workshop; California: La Jolla (2016).

[74] Vanoye CG Lossin C Rhodes TH George AL. Single-channel properties of human NaV1.1 and mechanism of channel dysfunction in SCN1A-associated epilepsy. J Gen Physiol. (2006) 127:1–14. doi: 10.1085/jgp.200509373 16380441 PMC2151481

[75] Mohler PJ Le Scouarnec S Denjoy I Lowe JS Guicheney P Caron L. Defining the cellular phenotype of “ankyrin-B syndrome” variants: human ANK2 variants associated with clinical phenotypes display a spectrum of activities in cardiomyocytes. Circulation. (2007) 115:432–41. doi: 10.1161/CIRCULATIONAHA.106.656512, PMID: 17242276

[76] Mohler PJ Splawski I Napolitano C Bottelli G Sharpe L Timothy K. A cardiac arrhythmia syndrome caused by loss of ankyrin-B function. Proc Natl Acad Sci USA. (2004) 101:9137–42. doi: 10.1073/pnas.0402546101, PMID: 15178757 PMC428486

[77] Schlipp A Schinner C Spindler V Vielmuth F Gehmlich K Syrris P. Desmoglein-2 interaction is crucial for cardiomyocyte cohesion and function. Cardiovasc Res. (2014) 104:245–57. doi: 10.1093/cvr/cvu206, PMID: 25213555

[78] Cordeiro JM Perez GJ Schmitt N Pfeiffer R Nesterenko VV Burashnikov E. Overlapping lqt1 and LQT2 phenotype in a patient with long QT syndrome associated with loss-of-function variations in KCNQ1 and KCNH2. Can J Physiol Pharmacol. (2010) 88:1181–90. doi: 10.1139/Y10-094, PMID: 21164565 PMC3076201

[79] Capell BC Collins FS. Human laminopathies: nuclei gone genetically awry. Nat Rev Genet. (2006) 7:940–52. doi: 10.1038/nrg1906 17139325

[80] Perrot A Hussein S Ruppert V Schmidt HHJ Wehnert MS Duong NT. Identification of mutational hot spots in LMNA encoding Lamin a/C in patients with familial dilated cardiomyopathy. Basic Res Cardiol. (2009) 104:90–9. doi: 10.1007/s00395-008-0748-6, PMID: 18795223

[81] Hu D Barajas-Martinez H Pfeiffer R Dezi F Pfeiffer J Buch T. Mutations in SCN10A are responsible for a large fraction of cases of brugada syndrome. J Am Coll Cardiol. (2014) 64:66–79. doi: 10.1016/j.jacc.2014.04.032, PMID: 24998131 PMC4116276

[82] Shorvon S Tomson T. Sudden unexpected death in epilepsy. Lancet. (2011) 378:2028–38. doi: 10.1016/S0140-6736(11)60176-1 21737136

[83] Hitiris N Suratman S Kelly K Stephen LJ Sills GJ Brodie MJ. Sudden unexpected death in epilepsy: a search for risk factors. Epilepsy Behav. (2007) 10:138–41. doi: 10.1016/j.yebeh.2006.11.010 17196884

[84] Feng HJ Faingold CL. Abnormalities of serotonergic neurotransmission in animal models of SUDEP. Epilepsy Behav. (2017) 71:174–80. doi: 10.1016/j.yebeh.2015.06.008, PMID: 26272185 PMC4749463

[85] Petrucci AN Joyal KG Purnell BS Buchanan GF. Serotonin and sudden unexpected death in epilepsy. Exp Neurol. (2020) 325:113145. doi: 10.1016/j.expneurol.2019.113145, PMID: 31866464 PMC7029792

[86] Bhandare AM Kapoor K Farnham MMJ Pilowsky PM. Microglia PACAP and glutamate: friends or foes in seizure-induced autonomic dysfunction and SUDEP? Respir Physiol Neurobiol. (2016) 226:39–50. doi: 10.1016/j.resp.2016.01.003, PMID: 26821120

[87] Troskie C. *Overlapping genetic risk in the spectrum of sudden death*. Vancouver (2016).

[88] Vezzani A Balosso S Ravizza T. Neuroinflammatory pathways as treatment targets and biomarkers in epilepsy. Nature reviews. Neurology. (2019) 15:459–72. doi: 10.1038/s41582-019-0217-x 31263255

[89] Hindocha N Nashef L Elmslie F Birch R Zuberi S Al-Chalabi A. Two cases of sudden unexpected death in epilepsy in a GEFS+ family with an SCN1A mutation [1]. Epilepsia. (2008) 49:360–5. doi: 10.1111/j.1528-1167.2007.01439_2.x, PMID: 18251839

[90] FbA N Borlot F Cossette P Minassian BA Andrade DM. Two definite cases of sudden unexpected death in epilepsy in a family with a DEPDC5 mutation. Neurology. Genetics. (2015) 1:28. doi: 10.1212/NXG.0000000000000028 PMC4811380 27066565

[91] Møller RS Larsen LHG Johannesen KM Talvik I Talvik T Vaher U. Gene panel testing in epileptic encephalopathies and familial epilepsies. Mol Syndromol. (2016) 7:210–9. doi: 10.1159/000448369, PMID: 27781031 PMC5073625

