# Supplementary material for: Cardiac arrhythmia and epilepsy genetic variants in sudden unexpected death in epilepsy
Source: Front Neurol. 2024 May 2;15:1386730. doi: 10.3389/fneur.2024.1386730 (PMC11097959; doi:10.3389/fneur.2024.1386730)
Supplement: Supplementary file 1 [file Table_1.docx]

Supplement Table 1. Genes currently included in the GeneDX Comprehensive Epilepsy Panel and Comprehensive Arrythmia panel. Genes identified in this cohort are in bold.

| Comprehensive Epilepsy Panel Genes (144 genes) | Comprehensive arrythmia panel Genes (58 genes) |
| --- | --- |
| *ADSL, ALDH5A1, ALDH7A1, ALG13, ANKRD11, ARG1, ARHGEF9,* ***ARX****, ASNS, ATP1A2, ATP1A3, ATP6AP2, ATRX, BRAT1, C12orf57,* ***CACNA1A****, CACNA1E, CACNA1G, CASK,* ***CDKL5****,* ***CHD2****,* ***CHRNA2****,* ***CHRNA4****, CHRNA7, CHRNB2, CLCN4, CLN3, CLN5, CLN6, CLN8, CNTNAP2, CSTB,* ***CTSD****, CTSF, CUL4B, DCX, DDX3X, DEPDC5, DNAJC5, DNM1, DOCK7, DYRK1A, EEF1A2, EHMT1,* ***EPM2A****, FGF12, FLNA, FOLR1, FOXG1, FRRS1L, GABBR2, GABRA1,* ***GABRB2****, GABRB3, GABRG2, GAMT, GATM, GLDC, GNAO1,* ***GOSR2****, GRIN1, GRIN2A,* ***GRIN2B****, HCN1, HNRNPU, IQSEC2, KANSL1, KCNA2, KCNB1, KCNC1, KCNH1, KCNJ10, KCNMA1, KCNQ2, KCNQ3, KCNT1, KCTD7, KDM6A, KIAA2022 (NEXMIF), LGI1, MAGI2, MBD5, MECP2, MEF2C,* ***MFSD8****, NALCN, NGLY1, NHLRC1, NPRL3, NR2F1,* ***NRXN1****, PACS1, PAFAH1B1, PCDH19, PHGDH,* ***PIGA****, PIGG, PIGN,* ***PIGO****, PIGT,* ***PIGV****, PLCB1,* ***PNKP****, PNPO,* ***POLG****, PPP2R5D, PPT1, PRRT2, PURA, QARS, SATB2,* ***SCARB2****,* ***SCN1A****, SCN1B,* ***SCN2A****, SCN8A, SHANK3,* ***SLC13A5****, SLC19A3,* ***SLC25A22****, SLC2A1, SLC35A2, SLC6A1, SLC6A8, SLC9A6, SMARCA2, SMC1A, SNAP25, SPATA5, SPTAN1, STX1B, STXBP1, SYNGAP1, SZT2,* ***TBC1D24****, TBL1XR1, TCF4,* ***TPP1****, TSC1, TSC2, TUBB2A, UBE3A, WDR45,* ***WWOX*** | *ABCC9, AKAP9,* ***ANK2****,* ***CACNA1C****, CACNA2D1, CACNB2, CALM1, CALM2, CALM3, CASQ2, CAV3, CTNNA3, DES,* ***DSC2****,* ***DSG2****, DSP, FLNC, GATA4, GATA5, GATA6, GJA5, GNB5, GPD1L, HCN4,* ***JUP****, KCNA5, KCND3,* ***KCNE1****, KCNE5, KCNE2, KCNE3, KCNH2, KCNJ2, KCNJ5,* ***KCNJ8****,* ***KCNQ1****, LDB3,* ***LMNA****, MYL4, NKX2-5, PKP2, PLN, PPA2,* ***RANGRF****,* ***RYR2****,* ***SCN10A****, SCN1B,* ***SCN2B****, SCN3B, SCN4B,* ***SCN5A****,* ***SNTA1****, TECRL,* ***TGFB3****, TMEM43,* ***TRDN****,* ***TRPM4****,* ***TTN*** |

Supplemental Table 2. Protein expression summary of Variants of Interest.

| Epilepsy genes | Organ(s) |
| --- | --- |
| *CACNA1A* | High expression in CNS; gastrointestinal tract; liver; smooth muscle; tonsil |
| *CDKL5* | Nuclear and cytoplasmic expression in most tissue, including heart muscle and CNS. |
| *EPM2A* | CNS; heart muscle; rectum; liver; kidney; secretory system; ovary; testis |
| *GRIN2B* | High expression in CNS |
| *MFSD8* | Cytoplasmic expression in most tissue, including heart muscle and high expression in CNS |
| *SCN1A** | High expression in CNS |
| *SCN2A* | High expression in CNS; kidney |
| Cardiac genes |  |
| *DSG2* | Cytoplasmic and membranous expression in essentially all cells except in lymphoid tissue and the CNS. |
| *KCNE1** | Heart muscle; lung; kidney; testis; ovaries; small intestine; peripheral blood leukocytes |
| *RYR2* | Cytoplasmic expression in heart muscle; CNS; placenta; testis |
| *SCN5A** | Primarily expressed in heart muscle; CNS |
| *SCN10A** | Heart muscle; CNS |

* Complete protein expression and localization profile not available
